# Exploring the third delay: an audit evaluating obstetric triage at Mulago National Referral Hospital

**DOI:** 10.1186/s12884-016-1098-2

**Published:** 2016-10-10

**Authors:** Jennifer Forshaw, Stephanie Raybould, Emilie Lewis, Mark Muyingo, Andrew Weeks, Kate Reed, Logan Manikam, Josaphat Byamugisha

**Affiliations:** 1 University of Liverpool School of Medicine, Liverpool, UK; 2 Eleanor Bradley fellow, Liverpool-Mulago Partnership for Women’s and Children’s Health, University of Liverpool, Liverpool, UK; 3 Department of Obstetrics and Gynaecology, Mulago Hospital, Makerere University College of Health Sciences, Kampala, Uganda; 4 International Women’s Health, University of Liverpool, Liverpool, UK; 5 Guy’s King’s & St Thomas’ School of Medical Education, King’s College London, London, UK; 6 Institute of Child Health, niversity College London, London, UK

**Keywords:** Obstetric, Triage

## Abstract

**Background:**

Mulago National Referral Hospital has the largest maternity unit in sub-Saharan Africa. It is situated in Uganda, where the maternal mortality ratio is 310 per 100,000 live births. In 2010 a ‘Traffic Light System’ was set up to rapidly triage the vast number of patients who present to the hospital every day.

The aim of this study was to evaluate the effectiveness of the obstetric department’s triage system at Mulago Hospital with regard to time spent in admissions and to identify urgent cases and factors adversely affecting the system.

**Methods:**

A prospective audit of the obstetric admissions department was carried out at the Mulago Hospital. Data were obtained from tagged patient journeys using two data collection tools and compiled using Microsoft Excel. StatsDirect was used to compose graphs to illustrate the results.

**Results:**

Informal triage was occurring 46 % of the time at the first checkpoint in a woman’s journey, but the ‘Traffic Light System’ was not being used and many of the patient’s vital signs were not being recorded.

**Conclusions:**

It is hypothesised that the ‘Traffic Light System’ is not being used due to its focus on examination finding and diagnosis, implying that it is not suitable for an early stage in the patient’s journey. Replacing it with a simple algorithm to categorise women into the urgency with which they need to be seen could rectify this.

## Background

Uganda has a population of over 35 million and a gross national per capita income of $490 [1]. Despite its turbulent neighbours, Uganda is relatively stable; its current government has been in power since 1986 [2]. Most of the population is agrarian, yet the majority of medical care is focused in urban areas where only 13 % of the population resides [2]. Although the public healthcare system is government funded, it lacks resources and is overstretched [3]. Figures suggest there are 11.7 physicians per 100,000 inhabitants in Uganda compared to 280 per 100,000 in the United Kingdom [4]. Maternal health remains an ongoing challenge worldwide to healthcare professionals despite a focus on reducing maternal mortality in the United Nations Millennium Development Goals [5]. The World Health Organisation’s statistics from Uganda in 2010 indicate that the maternal mortality ratio (MMR) is 310 per 100,000 live births in comparison with the United Kingdom with an MMR of 12 per 100,000 [6]. The lifetime risk of maternal death in sub- Saharan Africa is 1 in 16; thus, it is vitally important to make efforts in order to reduce the number of fatalities [1].

The Liverpool Mulago Partnership is an independent registered charity set up between the largest maternity unit in England, Liverpool Women’s NHS Foundation Trust, and Mulago National Referral Hospital, the biggest maternity unit in sub-Saharan Africa. Its aim is to share resources and knowledge and provide training opportunities for both units [7]. Mulago Hospital delivered over 33,000 babies in 2011 and is situated in Kampala the capital city of Uganda [8]. In 2011, the MMR for Mulago Hospital was 576 deaths per 100,000 live births [7], which is significantly higher than the national average. This figure emphasises the importance of reducing maternal deaths in this particular unit. With many government units lacking resources and staff, Mulago Hospital presents a final option for many women with obstetric complications, both from Kampala and outside of the city. Given that it never rejects women presenting with complications, its facilities have become hugely overstretched. In addition, the number of patients attending the unit increases with an increase in its quality: in the last 10 years, the number of deliveries has increased from 24,000 to 31,000 (Andrew Weeks, personal communication). The logistics of dealing with such massive numbers is daunting and new ways to organise care are needed.

The concept of triage describes sorting patients in order of the urgency of their condition, prioritising those who require prompt treatment. It is done by rapidly assessing all patients to determine their immediate medical need [9]. Triage is an important tool in ensuring that patients have access to care within an appropriate time frame, and thus is a key factor in reducing mortality [10, 11]. Western studies have recognised the importance of obstetric triage in reducing maternal mortality [12–14], but the evidence base for Uganda and other low and middle income countries is limited. Due to the high volume of patients at Mulago hospital, triage could be a particularly valuable tool for the maternity department.

The Eleanor Bradley Fellowship is a scheme by which successive British obstetricians are selected to work in Mulago Hospital for a year at a time [15]. During the second year of this programme, a ‘Traffic Light System’ was developed and introduced to the labour ward by the fellow in collaboration with the Mulago specialists (Appendix 1). This system is based on that of UK units and uses the patients’ presenting complaint, observations, results of basic tests such as urine dipstick, past medical and obstetric history, and diagnosis to assign a category to them. Depending on the classification there is a recommended time frame and provider assigned to the patient. The Manchester Triage System [16] is used to recommend the time frame, and the ‘Traffic Light System’ is used to identify who should review the patients.

The Mulago admissions system works as follows: patients enter the department at ‘*I*’ and approach the admissions desk marked ‘*II*’. At the desk, a file is created for the patient and the patient is asked to wait on the benches ‘*III*’ outside the admissions room ‘*IV*’. Women are then called into the admissions room one by one and examined.

Triage is possible at two steps along this journey: one being at the admissions desk (*II*), which would affect the order in which patients are called into the admissions room, and the second being in the admissions room itself (*IV*).

Two years have passed since this ‘Traffic Light System’ was first introduced. The following audit was designed to look at how the system is being used and to identify any problems with either the system itself or its implementation. The aim of this process is to ensure that the system provides maximum benefit, particularly as there is no recent data about whether or not the system is being consistently applied.

The audit looked at the time taken for women to pass through the triage and assessment system, whether they were categorised using the ‘Traffic Light System’ and whether they were seen within the recommended time frame for their category. It also recorded what observations were made, the diagnosis and the cause of any delays or adverse outcomes.

The aims of our study were: (1) to determine the time taken from entry into the department to first assessment by a healthcare professional, as well as the timing of each step leading to this point (2) to identify any common delays in the women’s journeys and any adverse consequences caused due to this (3) to assess whether the ‘Traffic Light System’ is used consistently for each woman and whether it is suitable for the department and to identify any problems with the tool itself or its implementation (4) to compare the time taken for a woman to be seen by a healthcare professional using the ‘Traffic Light System’, with the recommended time set out by the Manchester Triage System (5) to identify and evaluate any other strategies that allow some women to be prioritised over others (6) to follow up on the Mulago Hospital’s Maternity Department’s recommendations following presentation of the audit results (7) to provide a method for re-auditing the triage system once any recommendations have been put in place.

## Methods

As patients entered the department, they were given a slip of paper by the guard at the entrance with an identifier and the time they entered the department. All patients then went to the admissions desk where the identifier was written on the corner of their notes. At this point, ‘Data Collection Tool One’ (Appendix 2: Table 3) was used to record the time their file was created and any observations or triage that was completed. Once the patient entered the admissions room, a second researcher used ‘Data Collection Tool Two’ (Appendix 3: Table 4) to record the time they entered the admissions room, the time they were first assessed and other details about the patient’s condition. These collection tools were then paired up, using the patient identifiers, to show a complete patient journey through the admissions system. Study subjects were only included in the data collection for analysis if they completed the patient journey to the admission room.

Data was collected in 12 h shifts over a 10-day period, including weekend days and one night shift, to create a comprehensive picture of triage usage. The data collection required the presence of two researchers and a guard at the department entrance. Due to the limited number of researchers collecting data and the restriction of a 12 h shift, only 98 patient journeys were collected despite the unit receiving approximately 700 pregnant women during the study time.

After all the raw data had been obtained, it was compiled onto Microsoft Excel spreadsheets and analysis was conducted using statistical formulas on Excel. Graphs to represent the data were created using Microsoft Excel and StatsDirect.

## Results

A total of 98 completed patient journeys through the admissions department were recorded.

### Demographics

The mean age of patients was 24 years with primigravida being the most common parity. We found that 64 % of patients were booked, meaning they had received antenatal care. 29 % self presented with no prior antenatal care and were therefore unknown to the hospital. The remaining 7 % of the patients were referred, with the most common reason for presentation in both the referred and self-presenting patients being ‘labour-like pains’.

### Timing

The longest waiting period was between the patient entering the department and their file being created (Table 1). The average length of time from entering the department to assessment in the admissions room was 194 min (Table 1). All stages of the journey took longer at night, 8 pm to8 am, with the longest wait times between 6 am to 7 am. The time from entering the department to first being seen by a HCP decreased from 192 min to just 38 min when a midwife was present at the admissions desk (Table 2).

**Table 1:** Average time taken at each stage of the women’s journey

Step of the Journey	Average Time Taken (h:min)
Enter department to file creation (A to B)	01:27
File creation to admissions room (B to C)	01:02
Enter admissions to assessment (C to D)	00:56
Enter department to seeing healthcare professional (HCP) (A to seeing HCP)	02:53
Enter department to being assessed in admissions (A to D)	03:14

**Table 2:** Time taken to see healthcare professional

Midwife	Frequency	Average time until seen by HCP (h:min)
Present on desk	10	00:38
Not present	98	03:12

We found that 9 of the 98 patients were accompanied by or were friends with a member of staff. The mean time taken at every stage of their journey was less than the average for unaccompanied patients. The mean wait for those brought to the attention of a healthcare professional (HCP) was also greatly reduced.

### Triage

**Fig. 1:**
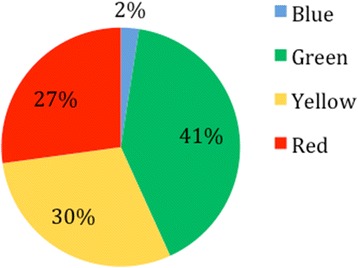
Category to which patients would have been assigned if the ‘Traffic Light System’ had been used

**Fig. 2:**
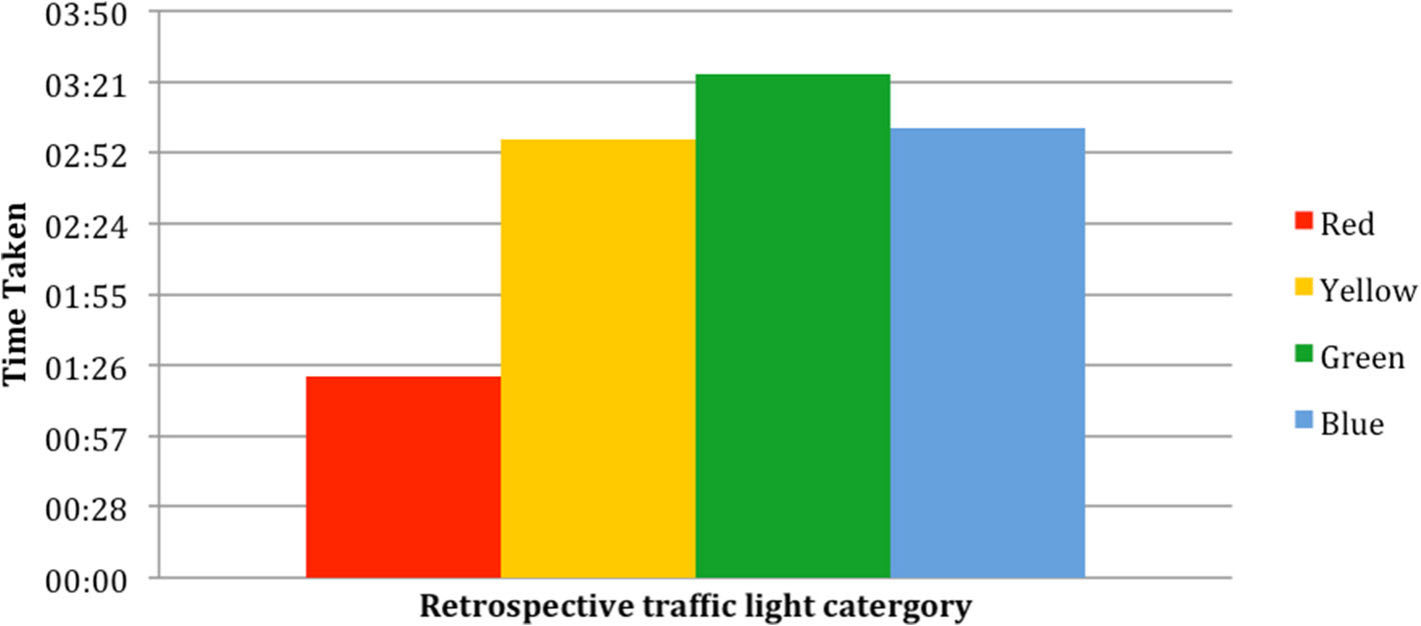
Average total journey time for each group, categorised retrospectively

We found that 46 % of the patients were informally triaged at the admissions desk. None of these patients were given a category as per the ‘Traffic Light System’. When patients were retrospectively categorised, green was the most common group (41 %), followed by yellow (30 %), red (27 %) and blue (2 %) (Fig. 1). Although they were not formally grouped, those who would have been categorised as red were seen fastest, followed by yellow, with green and blue patients taking the longest time to be seen (Fig. 2).

### Observations conducted in the assessment room

The most common vital sign measured was the patient’s blood pressure, which was assessed in 47 % of patients in the admissions room. The foetal heart rate was auscultated 55 % of the time, while 19 % of the time it was not applicable. Other observations were very rarely completed.

### Effect of known delays

**Fig. 3:**
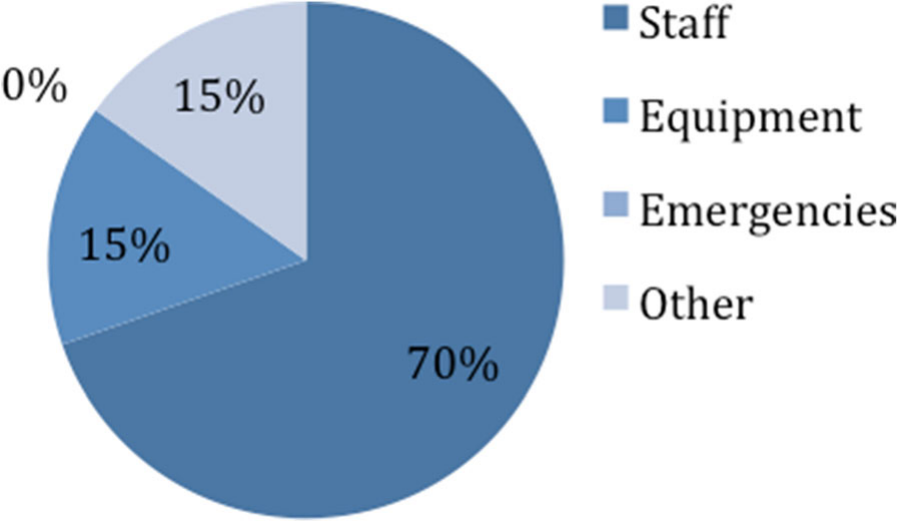
Reason for delay that was recorded in the 33/98 women where a specific reason was stated

For patients with a recorded reason for delay (33 %), the mean wait and, in particular, the maximum journey times were greatly increased. Of the recorded delays, 70 % were due to lack of staff (Fig. 3).

### Management in the assessment room

We found that 7 % of those assessed in the admissions room had an immediate investigation, all of which was urinalysis. We also found that 8 % received immediate management, including oxytocin or artificial rupture of membranes (AROM).

## Discussion

### Referrals

Of the 25 referred patients, 44 % were for ‘labour-like pains’ with potential cause for complication, such as the mother being a primigravida. This was deemed a risk factor as it was unknown if the woman’s pelvis would be favourable for labour and there was a higher risk of morbidities such as pre-eclampsia. The smaller maternity units’ low threshold for referral to Mulago increases the pressure on the maternity unit at Mulago by pushing even more patients through the doors.

The high level of referral and associated travel time could help explain the 2011 Mulago Hospital Maternal Mortality ratio of 576 per 100,000 live births [6], which is significantly higher than the national average.

### Patient observations

Patients’ vital signs were often only taken if the doctors had a reason to be concerned. For example, blood pressure was taken if the mother had oedema or was a primigravida and thus more likely to have pre-eclampsia. Part of the reason for this was the time taken to fetch a sphygmomanometer, as readings were more likely to be taken when a cuff was already present. The lack of equipment does not explain the lack of pulse or respiratory measurements. These seemed to be viewed as less important by staff working under significant time restraints.

### Timing

The most significant observation was the difference in time taken to be seen depending on the time of day the patient entered the department. Journey times were considerably less from 10 am to 5 pm, increasing sharply after 2 am. This may be especially important given that the patients who present through the night are likely to be more unwell and less able to wait until morning for care. The increased times are most likely due to less staff being present on the wards during night shifts (Fig. 3). Although the day staff are present in the hospital from 8 am, the journey times tend not to improve until later in the morning, possibly due to the large volume of staff who attend the morning meeting.

### Presence of a midwife on the desk

As expected, the presence of a midwife at the admissions desk significantly decreases the time taken for a patient to be first seen by a HCP (Table 2).

#### Triage

Results show that informal triage is completed in 46 % of cases and occurs at the admissions desk and not in the room. Informal triage consists of questioning regarding bleeding, previous caesarean sections, and referral. Those patients who answer positively to any question or who are in obvious distress move higher up the queue and enter the admissions room sooner. Evidence of the presence of informal triage is shown by the journey times of patients decreasing with increasing severity of their condition (retrospectively calculated; Fig. 2). This shows that although triage is not systemically carried out, it does occur. This has a measurable effect on the waiting times of patients.

None of the population studied was allocated according to the Traffic Light System. There may be many reasons for this but the most likely seemed to be that patients were triaged at the admissions desk prior to diagnosis. This is not an appropriate environment for examination, meaning that the Traffic Light System is often not suitable as it uses diagnoses or examination findings to categorise patients.

The current practice of triaging at the admissions desk is seemingly the most appropriate option as the average wait before being examined in the admissions room (Table 1) is 194 min, which is too long to wait to be first triaged.

### Limitations

Although this study provides useful information, it also has its limitations. One of the most substantial is that the researchers only worked 12 h per day. Any patients who took longer to pass through the system were not counted because the research team were no longer on duty when they were assessed. These variable missing data items could not be substituted with an assumed value as there were no realistic ways to make such assumptions. Hence, longer cases may be underrepresented, and the data may show a disproportionate number of short cases as it was more likely that the completed journeys for these patients would be collected.

Other limitations include the fact that only one night shift was completed, and although this shows a statistically significant difference in waiting times to the day shifts, it may have been that this was not a typical night shift. Therefore, it is recommended that more data should be collected, particularly at night, to see if the differences found are reproducible.

As there was no central tracking system for patients once they left the admissions room, there was little follow-up. This meant that although immediate adverse outcomes were recorded on the data collection tools, later problems, such as stillbirths or eclamptic seizures, may have been missed. It is also difficult to say which of the negative outcomes would have occurred no matter how quickly the women were seen. So inversely, some negative outcomes that were attributed to the delay in assessment may have been inevitable.

It must also be considered that the presence of researchers may have affected the results (Hawthorne Effect) [17], although the extent or nature of such an impact cannot be stated with any certainty. For example, the presence of researchers could have prompted the staff to triage the patients more quickly than usual or to be more thorough in their assessment. In addition, there may have been a behavioural change in the staff such as less preferential treatment to certain patients. Inversely, the presence of a researcher by the admissions desk may have made staff presume they were doing clinical work and thus delay their own assessment and triaging. Furthermore, patients may have altered their behaviour due to the presence of researchers such as being more apprehensive about approaching the admissions desk or expecting clinical attention from the external researchers. As it is impossible to state the magnitude or even the direction of this factor, it would be advisable to repeat the study over a much longer period of time using local staff or a mounted video camera to collect data. Increasing the sample size would hopefully decrease the effect of any bias due to habituation.

A further limitation of the study is the language barrier. Often, staff speak to patients in Luganda and so parts of the history or assessment may have been missed by English-speaking research staff. Although the researchers asked what the conversation entailed, it is likely that some details were not communicated. Again, a solution to this would be to repeat the study using local members of staff to collect the data.

### Recommendations

As previously mentioned, a key recommendation would be to repeat the research over a longer period of time using local staff. This study does, however, give some indications as to where and why the delays occur.

The fact that sphygmomanometers were not always available on the wards definitely contributed to delays in patients’ journeys as well as unrecorded blood pressures. It is likely that even a small injection of funds to ensure a sphygmomanometer on each ward would dramatically increase the number of patients who had their blood pressure recorded and even prevent some delays.

Ideally, more staff would be employed or allocated to admissions, in particular to ensure that there is always a member of staff present in the admissions room at night and during shift changes. Increasing staff numbers would also ensure that a midwife could be specifically allocated to triage. However, taking into account the finite resources and staff of Mulago hospital, this may not be immediately possible and should thus be considered a long term goal [18].

At the moment triage is informal, as discussed above. It usually occurs when midwives decide who to bring into the admissions room next. Triage should ideally happen as early as possible in a patient’s journey, with a rapid assessment that involves a brief history and examination. In the case of Mulago, this would mean that women were triaged in the admissions room, as there is a suitable space for a basic examination. However, due to volume of patients, this would mean that many patients would have to wait hours for triage, which is not a suitable option. Therefore, the most appropriate solution appears to be that women are assessed at the admissions desk using a brief history and observations, which would allow all women to be triaged early in their journey. Although observations at the desk do not suffice for a full examination, this process should still identify women who need to be seen more urgently [19]. As the admission desk is the most suitable place for this to occur, it may be appropriate to adapt the current Traffic Light System, and thus remove the need for examination or a diagnosis to categorise people.

As permanent staff in Mulago can best advice on changes to the Traffic Light System as well as other parts of triage, the following suggestions are designed to be fully discussed with, and adapted by, the staff at Mulago in order to ensure that the final ideas are suitable for their purpose.

One suggestion would be to implement the use of a systematic standardised flow diagram to categorise every patient. This would hopefully make the system simpler and ensure that every patient is triaged formally. An example of this sort of flow chart is shown in Figure 28. This draft was developed in discussion with local staff and Professor Weeks for adaptation by local staff.

One way to implement this system would be for the flow diagram to be made available for staff at the admissions desk who would write the assigned category and preliminary diagnosis on the patient’s notes. The benches outside the admissions room could then be colour-coded so that those working in the admissions room would know the category of each patient.

Ensuring the presence of a midwife at the admissions desk is a feasible expectation with the current level of staffing, as a midwife is often only called to the admissions room if someone is delivering. Hopefully, with the flow diagram system, those who are near delivery would proceed to the labour ward for assessment by the midwives. Thus, fewer births would occur in the admissions room, freeing up the midwife to triage patients. If this new flow diagram system was to be put in place it would be appropriate to re-audit in 3 months’ time to ascertain whether the new system was being used, whether or not it would be suitable and to provide an opportunity to highlight and correct any flaws.

A further long-term recommendation would be to continue the work that has been started by the latest Eleanor Bradley fellow to increase the number of deliveries that happen in smaller health centres, thus decreasing the number of referrals to Mulago and potentially alleviating some of the congestion.

## Conclusions

This short study provided a large amount of information. The key finding was that staff informally triaged, shortening the journey time for urgent patients. However, the ‘Traffic Light System’ was not being used and key observations such as vital signs were not taken. The admissions desk is the most appropriate place for triage to occur and there are elements of the ‘Traffic Light System’ that are not suitable for this setting. The suggested flow diagram and coloured bench system will be discussed with the staff at Mulago to produce consensus-based solutions. It is recommended to purchase several sphygmomanometers so that observations can be taken in the waiting area.

The majority of the study’s aims were met. However, it would be useful to repeat the data collection process over a longer period of time using local staff to remove some of the limitations and bias. In particular, patients were not followed up for long enough to fully assess the possibility of adverse consequences. If the flow diagram system is to be implemented to deal with some of the problems of the ‘Traffic Light System’, then a re-audit should be conducted in 3 months to evaluate its success.

## Appendix 1

Traffic Light Triage System

**Red:** (patient requires immediate senior medical review)

• Unable to talk/noisy breathing

• Respiratory rate of <5 or >25

• Pulse rate of >120 bpm

• Systolic blood pressure >160 mmHg

• Diastolic blood pressure >100 mmHg

• Unresponsive/unconscious patient

• Obvious obstetric emergency as outlined in primary survey

• Haemorrhage

• Severe abdominal pain

• Any woman with a history of seizure/blackout/faints

• Maternal fever >38.5 °C

• PROM with meconium or blood stained liquor

• Previous scar with abdominal pain

• Delay in second stage of labour

• Sickle cell crisis

• Referral from clinic/old Mulago with suspicion of foetal distress

**Yellow**: (patient requires medical assessment soon-JHO must consult SHO if in any doubt)

• Headache in the presence of proteinuria and mild hypertension

• Suspected preterm labour

• Absent foetal movements

• Maternal fever <38.5 °C

• PPROM

• PROM

• Referral with delay in first stage of labour

• Known multiple pregnancy in labour

• Known breech presentation in labour

• Previous scar in labour without scar tenderness

• Abdominal pain in pregnancy

• Suspected UTI: request urinalysis and manage as outpatient

**Green:** (Patient to be seen by JHO or treated by midwifery staff)

• Labouring woman at term with no other complications

• Reduced foetal movements (can be seen initially by midwife and USS arranged) and patient can be reviewed by the doctor after the scan

• Women in latent phase of labour at term/false labour: (if she has neither previous scar nor grand multipara nor PROM nor post-dates should be sent home and given return date)

**Blue:** (Patient to be seen by midwife on admission and referred to antenatal clinic)

• Oedema with normal blood pressure

• Varicose veins

• Haemorrhoids

• Suspected candida infection

• Genital warts

• Constipation

## Appendix 2

**Table 3:** Data to be collected at the admissions desk

Patient Number:
Time entered the department
Time seen by clerk at admissions
Time seen in triage
Time seen by healthcare professional (HCP)
Triage
Has the patient been referred? If so, from which institution?
Is the patient booked or un-booked?
Is the patient triaged?
If yes answer questions below:
Is the patient allocated a traffic light colour (red, orange, green, blue)?
Is the patient brought to the attention of a HCP quickly because of the seriousness of their condition?
Are the patient’s observation taken at triage?
Please record the observations (BP, P, RR, Temp, conscious level)
Is the foetus assessed? (FHR, foetal movements)
Diagnosis made at triage?
Is any treatment initiated at triage?
Additional factors affecting triage
Is the patient in obvious distress (crying, asking for help, in pain)?
Has the patient’s behaviour influenced how quickly they are seen?
Did the patient offer to pay a bribe?
Is the patient known by/accompanied by a member of staff?
If so did this influence how quickly they were seen?
Did the patient lose their place is the queue or get moved up the queue? If so, for what reason?

## Appendix 3

**Table 4:** Data to be collected in the admissions area (after triage)

Patient Number:	
Patient details	
Age and Parity	
Is the patient booked or un-booked for antenatal care at Mulago?	
Has the patient been seen previously for the same complaint?	
Nature of presenting complaint	
Gestation (weeks of amenorrhoea (WOA))	
Patient observations on assessment by HCP (BP, P, RR, Temp, conscious level)	
Foetal assessment by HCP (FHR, movements)	
Final diagnosis	
Immediate treatment given	
Are there any factors causing delay in initiation of treatment (i.e., lack of equipment, medicine or personnel)?	
Were there any immediate adverse outcomes (maternal death, IUFD, FSB)?	
Was senior help sought?	

## Abbreviations

AROM: Artificial rupture of membranes
HCP: Healthcare professional
MMR: Maternal mortality ratio
